# Effects of Dental Methacrylates on Oxygen Consumption and Redox Status of Human Pulp Cells

**DOI:** 10.1155/2014/956579

**Published:** 2014-02-12

**Authors:** Giuseppina Nocca, Cinzia Callà, Giuseppe Ettore Martorana, Loredana Cicillini, Sandro Rengo, Alessandro Lupi, Massimo Cordaro, Maria Luisa Gozzo, Gianrico Spagnuolo

**Affiliations:** ^1^Istituto di Biochimica e Biochimica Clinica, Facoltà di Medicina e Chirurgia, Università Cattolica del Sacro Cuore, Largo Francesco Vito 1, 00168 Rome, Italy; ^2^Laboratorio di Patologia Clinica, Ospedale M.G. Vannini, Via dell'Acqua Bullicante, 00177 Rome, Italy; ^3^Department of Neurosciences, Reproductive and Odontostomatological Sciences, University of Naples “Federico II”, Via G. Pansini 5, 80131 Napoli, Italy; ^4^Istituto di Chimica del Riconoscimento Molecolare, C.N.R., c/o Largo Francesco Vito 1, 00168 Rome, Italy; ^5^Istituto di Clinica Odontoiatrica, Facoltà di Medicina e Chirurgia, Università Cattolica del Sacro Cuore, Largo Francesco Vito 1, 00168 Rome, Italy

## Abstract

Several studies have already demonstrated that the incomplete polymerization of resin-based dental materials causes the release of monomers which might affect cell metabolism. The aim of this study was to investigate the effects of triethylene glycol dimethacrylate, 1,4-butanediol dimethacrylate, urethane dimethacrylate, and 2-hydroxyethyl methacrylate on (1) cellular energy metabolism, evaluating oxygen consumption rate, glucose consumption, glucose 6-phosphate dehydrogenase activity, and lactate production, and (2) cellular redox status, through the evaluation of glutathione concentration and of the activities of enzymes regulating glutathione metabolism. *Methods*. Human pulp cells were used and oxygen consumption was measured by means of a Clark electrode. Moreover, reactive oxygen species production was quantified. Enzymatic activity and glucose and lactate concentrations were determined through a specific kit. *Results*. Triethylene glycol dimethacrylate, 1,4-butanediol dimethacrylate, and 2-hydroxyethyl methacrylate induced a decrease in oxygen consumption rate, an enhancement of glucose consumption, and lactate production, whilst glucose 6-phosphate dehydrogenase and glutathione reductase activity were not significantly modified. Moreover, the monomers induced an increase of reactive oxygen species production with a consequent increase of superoxide dismutase and catalase enzymatic activities. A depletion of both reduced and total glutathione was also observed. *Conclusion*. The obtained results indicate that dental monomers might alter energy metabolism and glutathione redox balance in human pulp cells.

## 1. Introduction

Resin-based materials utilized in medicine largely in dentistry and also in orthopaedics [1, 2] are complex mixed materials consisting of an organic polymerizable matrix and an inorganic reinforcing filler coupled through a silanic agent [3, 4]. Resinous matrix is frequently composed of bisphenol A glycerolate dimethacrylate (Bis-GMA) with the addition of other methacrylic monomers whose main function is to improve the handling and get an easier incorporation of the filler. The most used compounds, because of their low viscosity, are triethylene glycol dimethacrylate (TEGDMA), 2-hydroxyethyl methacrylate (HEMA), urethane dimethacrylate (UDMA), and, occasionally, 1,4-butanediol dimethacrylate (BDDMA).

After performing dental restorations with the above described materials, small amounts of uncured monomers are released [5] into the oral cavity and—through dentinal diffusion [6–8]—in pulpal tissues, where monomers like HEMA and TEGDMA may reach millimolar concentrations [9], high enough to cause detrimental effects such as alteration of the cellular redox balance and other adverse biological effects [10]. Several researchers established that various dental monomers are able to cause potential damages to the oral soft tissues *in vivo* [11] and remarkable cytotoxicity effects* in vitro* [6–12]. In particular, the *in vitro* studies on TEGDMA, Bis-GMA, and HEMA showed that such monomers have genotoxic, allergenic [13], cytotoxic, estrogenic (mainly in the case of Bis-GMA and TEGDMA), and mutagenic activity and that they alter lipid metabolism, glutathione (GSH) concentration, reactive oxygen species (ROS) production, cell cycle, energy metabolism, and mitochondrial activity [14–22]. Moreover, TEGDMA suppresses heat shock protein 72 expression in human monocytes [23] and regulates glutathione transferase P1 activity [24], while HEMA reduces intracellular tyrosine phosphorylation [25]. UDMA induces cell cycle perturbation, ROS overproduction and GSH depletion in CHO-K1 cells [26].

Oxygen consumption rate is reputed to be a good marker of mitochondrial functionality: in fact the decrease of oxygen consumption capability causes the reduction of ATP production so that the cells have to boost anaerobic glycolysis to get ATP from sugars thus increasing glucose consumption and lactate production [20].

It has been reported that about 0.1–0.5% of molecular oxygen consumed during mitochondrial respiration is converted to ROS: some electrons can in fact, escape from the mitochondrial electron-transfer chain and react with O_2_ to form superoxide anion (O_2_^∙−^) [27–29] which, in turn, can be reduced to hydroxyl radical (OH^∙−^) and hydrogen peroxide (H_2_O_2_) through superoxide dismutase (SOD) catalytic activity [30]. H_2_O_2_ was further transformed into O_2_ and H_2_O by the activity of other enzymes like catalase and glutathione peroxidase [31]. When the levels of hydrogen peroxide are too low to activate catalase, the dismutation of such chemical species is carried out by the activation of glutathione peroxidase, an enzyme that needs GSH to perform its catalytic activity [31]. Enzymatic (i.e., dismutases, catalases and peroxidases) or nonenzymatic (i.e., vitamins A, C, and E and GSH) defensive systems are adopted by the cells against ROS because an increase of these chemical species inside the cells can induce oxidative alterations of biological macromolecules like proteins, lipids, and DNA with possible loss of their functions [32].

The present work was therefore carried out to evaluate the effects of subcytotoxic concentrations (i.e., values able to induce a mortality not higher than 20% in respect to control) of HEMA, TEGDMA, UDMA, and BDDMA on human dental pulp cells (HPCs). At first, possible alterations of cellular energy metabolism were considered evaluating oxygen consumption rate, glucose disposal, and lactate production. Subsequently, cellular redox status was examined determining ROS production, GSH concentration, and the activity of glucose 6-phosphate dehydrogenase (G6PDH), glutathione reductase (GR), superoxide dismutase (SOD), and catalase.

## 2. Materials and Methods

All chemicals and reagents were obtained from Sigma-Aldrich Srl, Milan, Italy, unless otherwise indicated.

### 2.1. Cell Culture

HPCs from healthy patient (obtained with informed consent and with approval from the Ethics Committee of the Catholic University) were used in this study. Culture was performed as previously described [33]. The tooth pulp tissue was cut into small pieces and incubated in phosphate buffered saline (PBS), containing type I collagenase (3 mg/mL) and dispase (4 mg/mL), for 60 min at 37°C. The cells were plated in tissue culture flasks (25 cm^2^) with Dulbecco's modified Eagles' medium (DMEM), supplemented with 10% fetal calf serum (FCS), L-glutamine (2 mmol/L), streptomycin (100.0 *μ*g/mL), and penicillin (1000 units/mL) at 37°C in humidified atmosphere (95% air, 5% CO_2_). The medium was replaced before the formation of cell monolayer. Cells at subconfluence, obtained with no more than 5 passages, were used in all experiments.

### 2.2. Preparation of Methacrylates Solutions

Stock dimethyl sulfoxide (DMSO) solutions of TEGDMA (from 0.2 mol/L to 3.0 mol/L), UDMA (from 0.05 mol/L to 0.2 mol/L), and BDDMA (from 0.1 mol/L to 0.4 mol/L) were prepared immediately before use. A final concentration of DMSO (0.1% v/v) was utilized in all samples because—as shown by preliminary studies—it did not induce any alterations in the parameters under study.

DMEM containing the monomers was then added to the exponentially growing HPCs at the following final concentrations: TEGDMA (3.0, 1.5, 0.7, 0.4, and 0.2 mmol/L), UDMA (0.2, 0.1, and 0.05 mmol/L) and BDDMA (0.4, 0.2, and 0.1 mmol/L).

DMSO was absent only in cells treated with HEMA because this monomer is hydrophilic and—therefore—it can be added purely to the medium to reach a final concentration ranging from 1.0 mmol/L to 8.0 mmol/L.

### 2.3. Cell Viability

Cytotoxic concentrations of TEGDMA, HEMA, UDMA, and BDDMA monomers were determined by the MTT (3-(4,5-dimethylthiazol-2-yl)-2,5-diphenyltetrazolium bromide) assay [34]. HPCs were seeded in a 96-well tissue culture dish at 8000 cells/well and, after 24 h of incubation, the medium was removed and the cell monolayer was incubated with the above indicated monomer concentrations for 24 h. The medium was replaced by a solution of MTT (0.5 mg/mL, 100 *μ*L/well) in PBS, and the cells were incubated at 37°C for 1 h in a 5% CO_2_ atmosphere. The MTT solution was replaced with DMSO (100 *μ*L/well) and gently swirled for 10 min. The optical density was measured by a plate reader at 540 nm (Packard Spectracount, Packard BioScience, Meriden, CT, USA). The results were expressed as the percentages of untreated cultures. Each experiment was performed five times in quadruplicate.

On the basis of the obtained results the highest concentration of each monomer inducing a decrease of succinate dehydrogenase (SDH) activity—less than 20% compared to control—was selected [35]. In order to confirm the obtained results and to evaluate cell number, the HPCs were treated as described above and the number of viable cells was determined by trypan blue exclusion test [36]. In this way the subcytotoxic concentration for each monomer was determined and used in all the following experiments.

### 2.4. Assays Condition

Exponentially growing HPCs (1.0 × 10^6^) in DMEM (20.0 mL) were incubated with each monomer for 4 and 24 h. On the basis of cellular toxicity results, all the experiments described below were performed with HEMA (4.0 mmol/L), or TEGDMA (0.7 mmol/L), or UDMA (0.2 mmol/L), or BDDMA (0.4 mmol/L), or DMSO 0.1%, or culture medium (control) and the incubations were performed in a humidified atmosphere of 5% CO_2_.

#### 2.4.1. Metabolic Assays

*(a) Oxygen Consumption Rate*. HPCs were incubated with the monomers, washed with PBS solution without Ca^++^ or Mg^++^, counted by trypan blue exclusion test, resuspended in Krebs Ringer Phosphate (KRP) buffer (1.0 × 10^6^ cells/mL) and finally utilized to monitor the oxygen consumption rate under constant stirring, for 10 min at room temperature (Oxygen Meter Model 781, Strathkelvin Instruments, Glasgow, UK). Results are expressed as percentage of oxygen consumption rate of treated cells versus control.

*(b) Determination of Cellular Glucose Consumption*. HPCs were incubated with the monomers; then the cellular protein content was determined through BioRad Protein Assay, using bovine serum albumin as standard.

Cellular glucose consumption was measured in the culture supernatants with the appropriate reagent kit (glucose GOD-PAP, Roche Diagnostics). In order to normalize glucose consumption at different cell numbers, data were expressed as the ratio

(1) $$\begin{array}{c} \frac{\text{Glucose}\;\;\text{concentration}\;\;\left(\text{mg}/\text{dL}\right)}{\text{Cellular}\;\;\text{proteins}\;\;\left(\text{mg}\right)}. \end{array}$$

*(c) Determination of Cellular Lactate Production*. After the incubation with monomers, the lactate concentration was then determined in cell extracts: cellular pellets obtained after centrifugation (400 g, 5 min, 4°C) were washed in PBS solution and stored (−80°C). Cell lysates were centrifuged (20,000 g, 15 min, 4°C) and the collected supernatants were used to determine the protein content as already indicated.

Lactate concentration was determined with the appropriate reagent kit (Lactate, Roche Diagnostics). In order to normalize lactate production at different cell numbers, data (expressed as percent of control) were calculated as the ratio

(2) $$\begin{array}{c} \frac{\text{Lactate}\;\;\text{concentration}\;\;\left(\text{mg}/\text{dL}\right)}{\text{Cellular}\;\;\text{proteins}\;\;\left(\text{mg}\right)}. \end{array}$$

#### 2.4.2. Cellular Redox Status Assays

*(a) Glucose-6-Phosphate Dehydrogenase Activity*. HPCs were incubated with monomers; then the enzymatic activity was determined in cell extracts (as already indicated). G6PDH activity (measured as nmol/min/mg protein) was determined by means of the absorbance increase induced by the reduction of NADP^+^ to NADPH, at 340 nm [37].

*(b) ROS Production*. ROS production was measured using an apolar oxidation-sensitive fluorescent probe 2′,7′-dichlorodihydrofluorescein diacetate (H_2_DCF-DA). The latter readily diffuses into the cells, where it is enzymatically deacetylated by intracellular esterases to a polar nonfluorescent derivative trapped inside. In the presence of ROS, the probe is oxidized to 2′,7′-dichlorodihydrofluorescein (DCF); fluorescence levels depend on the intracellular ROS concentration. The cells were seeded in a 96-well microplate (5 × 10^3^ cells/well) for 24 hrs. The H_2_DCF-DA probe (10 mmol/L) was added for 20 min at 37°C in the dark. The cells were then washed with PBS and monomers were added (cells with DMEM were used as control). The H_2_DCF-DA probe was then added to all samples and the formation of DCF was at once fluorimetrically monitored using a Glomax Multidetection System fluorometer (Promega, Milan, Italy) at 490 nm excitation and 526 nm emission wavelengths, for 4 h [38].

The viable cells in the microplate wells were estimated using the MTT assay after each measurement. The amount of viable cells present in each well was unchanged after 6 h of incubation, both in presence and in absence (control) of monomers. Because H_2_DCF-DA can react directly with the hydroxyl radical but not with superoxide or H_2_O_2_, the ROS detected in the resin monomer-treated cells were probably hydroxyl radicals [39, 40].

*(c) Cellular Glutathione Determination*. After the incubation with monomers, HPCs were washed twice with PBS, resuspended in trichloroacetic acid (6%, 100.0 *μ*L), and immediately stirred. The lysed cells were centrifuged (20,000 g, 4 min) and the supernatants were used to establish GSH and GSH + GSSG (total glutathione) amount. GSH concentration was determined by Ellman method [41] and modified by Wataha et al. [42]: briefly the supernatant (40.0 *μ*L) was added to Na_2_HPO_4_ (0.30 mol/L, 80 *μ*L) and 5,5′-dithiobis[2-nitrobenzoic acid] (DTNB, 0.04% in 1% sodium citrate, 10.0 *μ*L). Absorbance was measured at 405 nm (Packard Spectracount, Packard BioScience Company, Meriden CT USA) reporting the results as the percentage of treated versus untreated cells. Total glutathione was estimated as follows: a freshly prepared NaBH_4_ aqueous solution (20.00 mg/mL, 40.0 *μ*L) was added to the supernatant (40.0 *μ*L) previously shaken with ethyl ether (120.0 *μ*L) to remove the lipophilic substances. After incubation of the mixture (40 min, 37°C), HCl (1 N, 37.5 *μ*L), acetone (40.0 *μ*L) and Tris buffer (1.0 mol/L, pH 8.5, 30.0 *μ*L) were added and an aliquot of the solution (150.0 *μ*L) was mixed with DTNB (0.04% in 1.0% sodium citrate, 10.0 *μ*L) to determine its concentration spectrophotometrically, as described above.

*(d) Glutathione Reductase Enzymatic Activity*. HPCs were incubated with the monomers. The enzymatic activity was then determined in cell extracts obtained as already indicated. The GR activity was determined according to Carlberg and Mannervik [43], briefly: GR reduces GSSG to GSH at the expenses of NADPH, whose disappearance can be checked at 340 nm. Enzyme activity was expressed as nmol/min/mg protein.

*(e) SOD Enzymatic Activity*. HPCs were incubated with one of the monomers and SOD enzymatic activity was then determined in cell extracts (obtained as already indicated); the cellular pellets obtained after centrifugation (400 g, 5 min, 4°C) were washed with PBS solution and stored at −80°C.

The enzymatic activity was measured (Packard Spectracount, Packard BioScience) using the appropriate SOD determination kit (19160 Fluka Analytical, Sigma-Aldrich, Milan, Italy). Enzymatic activity was measured as nmol/min/mg protein and expressed as percentage of the control group.

*(f) Catalase Enzymatic Activity*. After the incubation with monomers, the HPCs intracellular extracts (obtained as already indicated) were used to determine catalase activity by means of a Packard Spectracount (Packard BioScience) using the appropriate Catalase Assay kit (Sigma-Aldrich, Milan, Italy). Enzymatic activity was measured as nmol/min/mg protein and expressed as percentage of the control group.

### 2.5. Statistical Analysis

Data are expressed as the mean ± standard deviations (SD) of at least 5 different experiments performed in duplicate (*n* = 5). Statistical analysis was performed by ANOVA with Bonferroni's posttest, or Student *t*-test. A level of *P* < 0.05 was assumed significant.

## 3. Results

### 3.1. Cell Viability

**Figure 1:**
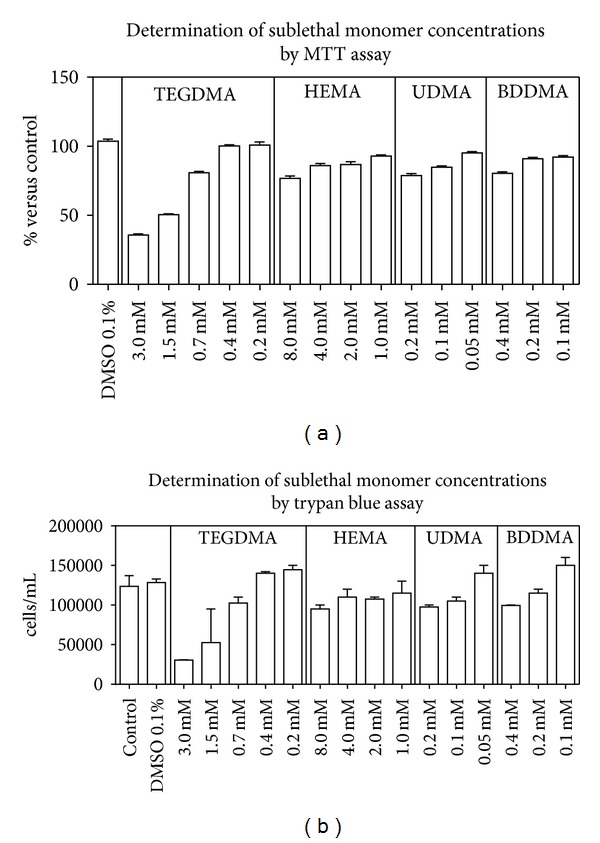
Evaluation of subcytotoxic concentrations. HPCs were treated with different concentrations of monomers for 24 h. The viability was determined by MTT assay (a) and the number of viable cells was evaluated by trypan blue exclusion test (b). On the basis of the obtained results, the highest concentration of each monomer inducing a decrease of cell numbers less than 20% was selected. MTT results were reported as percentage of viable cells versus control ± SD. Trypan blue data were reported as cell number in 1 mL of DMEM. Data are the mean of 5 different experiments performed in quadruplicates (*n* = 5).

As expected, all tested monomers caused a dose dependent decrease of cell vitality observed by MTT (Figure 1(a)); thus, subcytotoxic concentrations for each monomer were obtained. Results were confirmed using trypan blue exclusion test (Figure 1(b)).

Each monomer was then used at the following subcytotoxic concentrations: TEGDMA (0.7 mmol/L), HEMA (4.0 mmol/L), UDMA (0.2 mmol/L), and BDDMA (0.4 mmol/L).

### 3.2. Effects of HEMA, TEGDMA, UDMA and BDDMA on Examined Parameters

**Figure 2:**
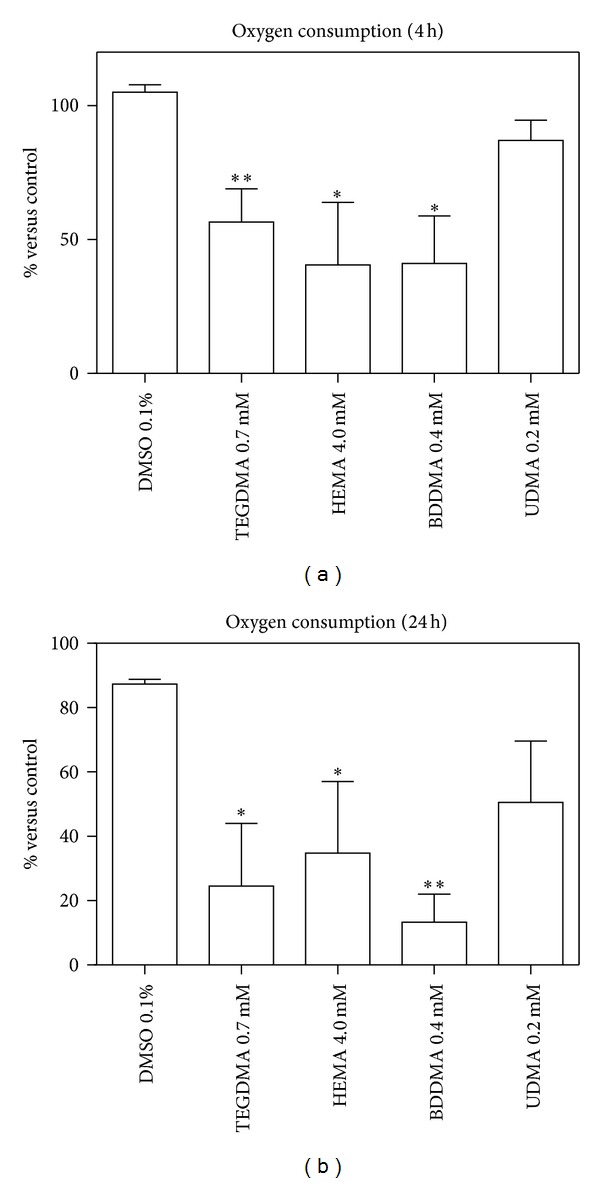
Oxygen consumption rate after cell treatment with HEMA (4.0 mmol/L), TEGDMA (0.7 mmol/L), BDDMA (0.4 mmol/L), or UDMA (0.2 mmol/L). The results were reported as percentage of oxygen consumption rate of treated cells versus control ± SD; *(*P* < 0.05) and **(*P* < 0.01) significantly different from DMSO treated cells. Data are the mean of 5 different experiments performed in duplicate (*n* = 5).

Mitochondrial function was monitored by determining respiration in HPCs suspensions. HEMA, TEGDMA, and BDDMA caused a significant decrease of cellular oxygen consumption rate versus cells treated with DMSO (Figure 2). In particular, after 4 h of incubation, the reduction is about 50%, whereas after 24 h O_2_ consumption was decreased to approximately 20% of DMSO treated cells (Figure 2).

**Figure 3:**
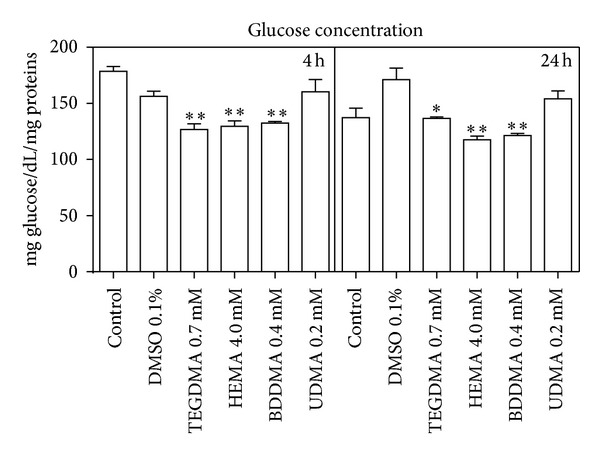
Glucose concentration in DMEM of HPCs untreated and treated with HEMA (4.0 mmol/L), TEGDMA (0.7 mmol/L), BDDMA (0.4 mmol/L), or UDMA (0.2 mmol/L); *(*P* < 0.05) and **(*P* < 0.01) significantly different from control. Data are expressed as the mean ± SD of 5 different experiments performed in duplicate (*n* = 5).

**Figure 4:**
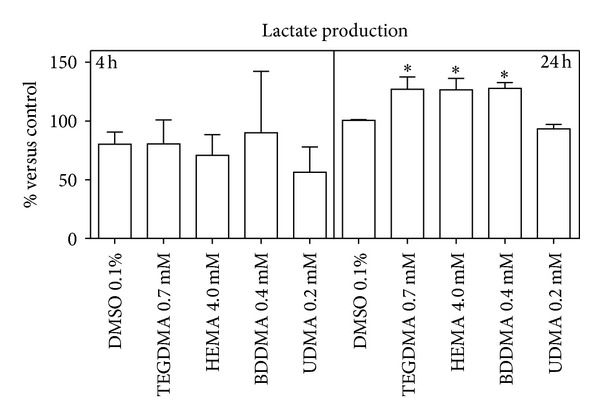
Lactate production by HPCs cells untreated and treated with HEMA (4.0 mmol/L), TEGDMA (0.7 mmol/L), BDDMA (0.4 mmol/L), or UDMA (0.2 mmol/L). The results were reported as percentages of lactate produced by treated cells versus control ± (SD); *(*P* < 0.05) significantly different from DMSO treated cells. Data are the mean of 5 different experiments performed in duplicate (*n* = 5).

**Figure 5:**
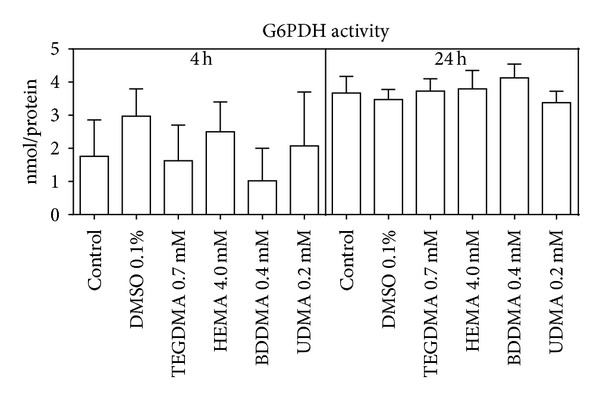
G6PDH activity of HPCs untreated and treated with HEMA (4.0 mmol/L), TEGDMA (0.7 mmol/L), BDDMA (0.4 mmol/L), or UDMA (0.2 mmol/L). Data are expressed as the mean ± SD of 5 different experiments performed in duplicate (*n* = 5).

**Figure 6:**
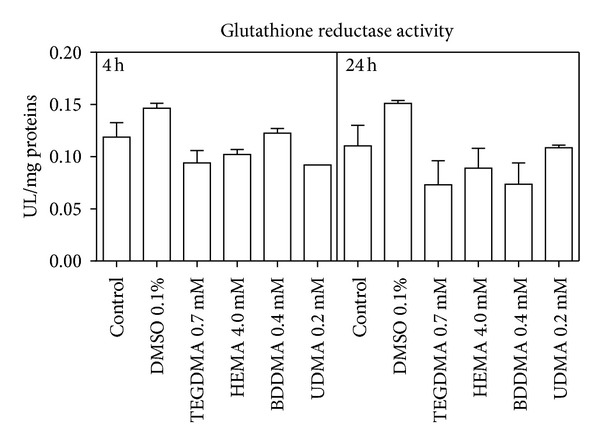
GR activity of HPCs untreated and treated with HEMA (4.0 mmol/L), TEGDMA (0.7 mmol/L), BDDMA (0.4 mmol/L), or UDMA (0.2 mmol/L). Data are expressed as the mean ± SD of 5 different experiments performed in duplicate (*n* = 5).

As a consequence of this alteration, the glucose consumption (Figure 3) and the lactate production (just after 24 h) (Figure 4) significantly increased, whilst G6PDH and GR activity (Figures 5 and 6) were not significantly modified with respect to control cells.

**Figure 7:**
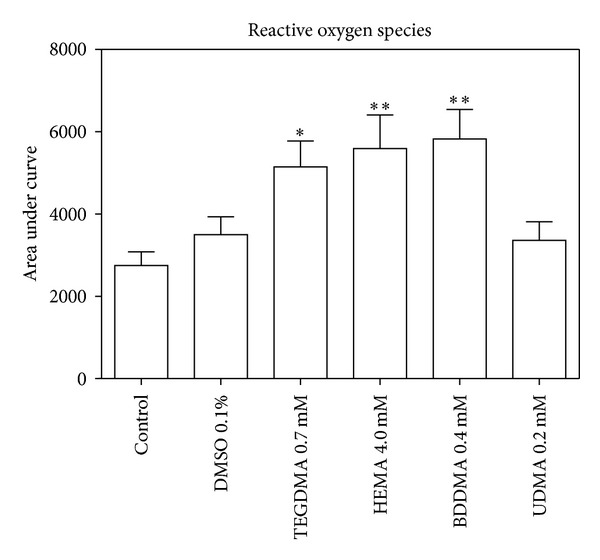
ROS produced by HPCs untreated and treated with HEMA (4.0 mmol/L), TEGDMA (0.7 mmol/L), BDDMA (0.4 mmol/L) or UDMA (0.2 mmol/L). *(*P* < 0.05) and **(*P* < 0.01) significantly different from control. Data are expressed as the mean ± SD of 6 different experiments performed in quintuplicate (*n* = 6).

**Figure 8:**
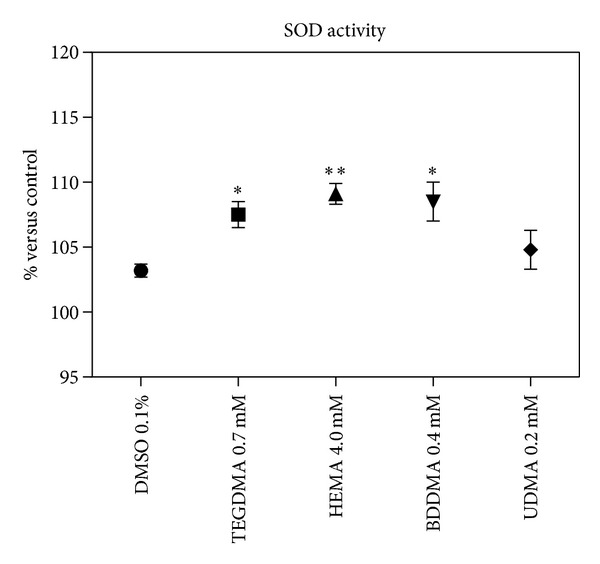
SOD activity of HPCs untreated and treated with HEMA (4.0 mmol/L), TEGDMA (0.7 mmol/L), BDDMA (0.4 mmol/L), or UDMA (0.2 mmol/L). The results were reported as percentage of enzymatic activity by treated cells versus control ± SD; *(*P* < 0.05) and **(*P* < 0.01) significantly different from DMSO treated cells. Data are the mean of 5 different experiments performed in triplicate (*n* = 5).

**Figure 9:**
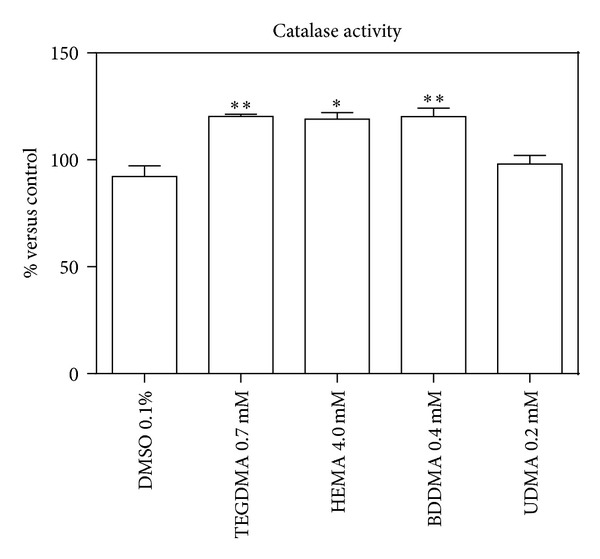
Catalase activity of HPCs untreated and treated with HEMA (4.0 mmol/L), TEGDMA (0.7 mmol/L), BDDMA (0.4 mmol/L), or UDMA (0.2 mmol/L). The results were reported as percentage of enzymatic activity of treated cells versus control ± SD; *(*P* < 0.05) and **(*P* < 0.01) significantly different from DMSO treated cells. Data are the mean of 5 different experiments performed in triplicate (*n* = 5).

To determine the role of ROS production in monomers toxicity, H_2_DCF-DA cellular oxidation was monitored. After 4 h of incubation, monomers caused an increase of ROS production (about 30–40%) in comparison with DMSO-treated cells (Figure 7). An increase of SOD (albeit slight) and catalase enzymatic activity, after 24 h, was consequently observed (Figures 8 and 9, resp.).

**Figure 10:**
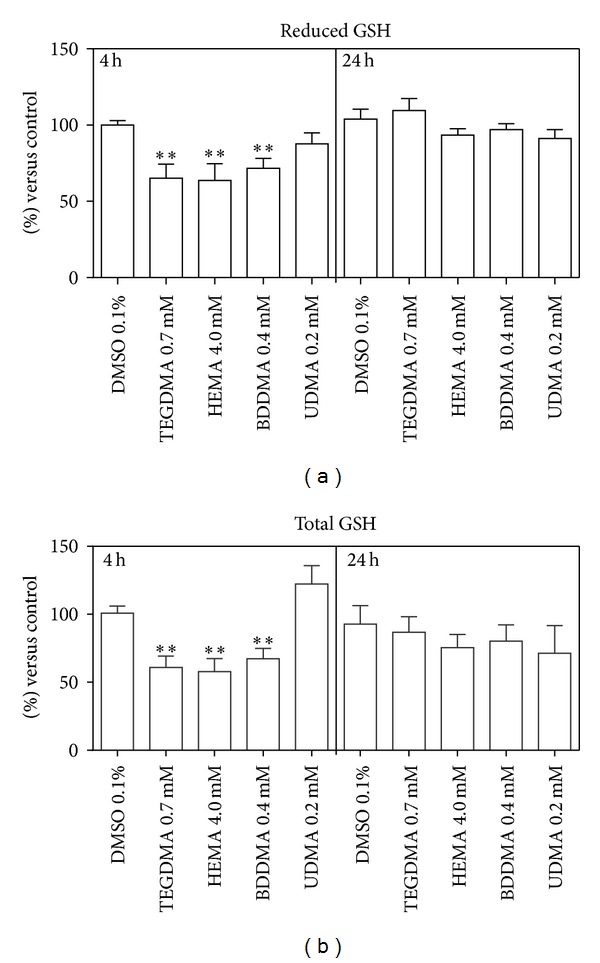
Concentration of reduced and total glutathione in HPCs untreated and treated with HEMA (4.0 mmol/L), TEGDMA (0.7 mmol/L), BDDMA (0.4 mmol/L), or UDMA (0.2 mmol/L). Results are expressed as percentage of treated cells versus control ± SD; *(*P* < 0.05) and **(*P* < 0.01) significantly different from DMSO treated cells. Data are the mean of 5 different experiments performed in duplicate (*n* = 5).

Moreover, TEGDMA, HEMA, and BDDMA induced a significant depletion of intracellular GSH after 4 h of incubation (about 30% of DMSO-treated cells) (Figures 10(a) and 10(b), left panel); such phenomenon completely disappears after 24 h incubation (Figures 10(a) and 10(b), right panel). Interestingly, no increase of intracellular GSSG occurred during this period. UDMA did not induce any alterations of examined parameters (Figures 2–10).

## 4. Discussion

Dental composite resins have been employed worldwide since the mid 1950s for adult and young patients: a careful evaluation of the interactions between the components of these materials and the host is therefore mandatory.* In vitro* tests are especially suitable for this purpose, allowing the independent assessment of the contributions of each resin component and the different metabolic aspects, whereas the same was not obtainable with *in vivo* trials [44]. Over the years, the biocompatibility concept evolved, taking into account not only the possible cytotoxic effects but also their underlying biochemical causes.

HPCs used in this study may get into direct contact with methacrylic monomers at concentrations capable of inducing alterations in several metabolic parameters. It is known that HEMA, TEGDMA, UDMA, and BDDMA can impair both energetic and redox metabolism in HL-60 cells [20, 21] and it was therefore of great interest to investigate the effect of these compounds also on HPCs which represent one of their potential cellular targets, especially in consideration of the multiple functions expleted by these cells, principally dentin formation and nutrition of the mineralized tissues.

For each monomer, subcytotoxic concentrations (i.e., values able to induce a decrease of vitality less than 20% of control) were selected by trypan blue and MTT cytotoxicity test to evaluate energy metabolism and redox status of the treated cells.

The experiments on the alterations of the energy metabolism were carried out by analyzing the cellular respiration rate, an index of mitochondrial functionality, by measuring the oxygen uptake and by monitoring the glucose consumption, which are all strictly connected parameters [45].

In this study, ROS production was analyzed using H_2_DCF-DA, a fluorescent probe readily oxidized by ROS.

Superoxide dismutase and catalase were tested because the activity of the former enzyme increases during oxidative stress, catalyzing the breakdown of superoxide radicals (thus providing the first line of defense against oxygen toxicity) [46], whereas the latter catalyzes the transformation of hydrogen peroxide into oxygen and water [47].

GSH redox metabolism was investigated, evaluating GR and G6PDH activity: the former enzyme is in fact NADPH dependent and catalyzes GSSG reduction whereas the latter regulates the rate of the hexose monophosphate (HMP) shunt [48], that is, the metabolic pathway producing NADPH [49] from glucose.

Glucose disposal was also evaluated by determining lactate production and G6PDH enzymatic activity. The increase of lactate production indicates that glucose is disposed of through anaerobic glycolysis to produce energy, whereas the increase of G6PDH activity suggests that the sugar is utilized to generate NADPH—in the hexose monophosphate shunt (HMP)—for the reduction of GSSG by GR catalysis [50, 51].

The obtained results showed that BDDMA, HEMA, and TEGDMA induced a decrease of oxygen consumption rate, a phenomenon probably due to an impairment of mitochondria and an increase of glucose consumption which involves, through anaerobic glycolysis, a consequent increase of ATP production in the cells. To confirm that glucose was consumed by glycolysis and not by HMP shunt, lactate production and G6PDH activity were evaluated. The experimental results showed that the three monomers induced an increase of cellular lactate production without alteration of G6PDH activity and that, consequently, glucose was disposed of mainly through anaerobic glycolysis to produce energy.

As expected, BDDMA, HEMA, and TEGDMA induced—even at subcytotoxic concentration—a decrease of total GSH (i.e., GSH + GSSG), which probably depends on the direct binding of the tripeptide to the monomers rather than to its oxidation, as already observed in previous papers [52–57]. In fact, the thiol group of GSH can bind, via a Michael addition reaction [58], the *α*,*β*-unsaturated carbon-carbon moiety of methacrylates causing the detoxification of these xenobiotics [24, 59]. The reaction is catalyzed by glutathione S-transferase (GST), one of several enzyme forms belonging to a multigene family involved in detoxification processes [60–62].

Data regarding the enzymatic activity of GR and G6PDH further confirm the above considerations: in fact, the activity of both enzymes is unaffected by monomers because the GSSG concentration is unchanged.

As already reported in literature [17–21], BDDMA, TEGDMA and HEMA provoked an overproduction of ROS and it is interesting to note that the observed decrease of oxygen consumption rate may provide an important indication about the source of radicals. As a matter of fact, the mitochondrial respiratory chain is a major site of ROS production inside cell and, when the rate of electron flow (coupled with oxygen consumption) is slow, electrons accumulated in the respiratory chain increase the reduction state of the electron transport chain components and, consequently, ROS formation [30].

A 24 h cell incubation with HEMA, TEGDMA, and BDDMA caused, in the reported experimental conditions, an increase of the enzymatic activity of SOD (albeit slightly) and catalase, subsequent to the increased ROS production (observed after 4 h).

In the reported experimental conditions UDMA did not cause alterations of the examined parameters, probably because its concentration was too low.

## 5. Conclusion

All the above-described results seem, therefore, to draw the following overview: TEGDMA, HEMA, and BDDMA alter mitochondrial function triggering two types of correlated events.

1. Increase of glucose consumption through glycolysis to produce ATP. In fact, enzymes involved in the hexose monophosphate shunt do not increase their activity while lactate production is increased.
2. Increase of ROS production (hydroxyl radicals that are derived from superoxide and peroxide) which determines the activation of SOD and catalase in order to protect the cells. The catalase activation was also caused by GSH lack and by the consequent impossibility of glutathione peroxidase to catalyze the tripeptide oxidation to counteract the increase of peroxide. On the other hand, an activation of glutathione peroxidase should have led to an increase of GR and G6PDH activity not actually observed.

The present study thus clearly demonstrates that, also at subcytotoxic concentration, HEMA, TEGDMA, and BDDMA might affect the mitochondrial activity by inducing alterations in energy metabolism, oxidative stress, and GSH balance in HPCs.
